# Reliability of Morphological and PCR Methods for the Official Diagnosis of *Aethina tumida* (Coleoptera: Nitidulidae): A European Inter-Laboratory Comparison

**DOI:** 10.3390/insects13010033

**Published:** 2021-12-28

**Authors:** Stéphanie Franco, Nicolas Cougoule, Amandine Tison, Aurélie Del Cont, Cristina Gastaldi, ILC Consortium, Véronique Duquesne

**Affiliations:** 1 Honey Bee Pathology Unit, European Union Reference Laboratory for Bee Health, French Agency for Food, Environmental and Occupational Health and Safety (ANSES), 06902 Sophia Antipolis, France; nicolas.cougoule@anses.fr (N.C.); amandine.tison@anses.fr (A.T.); aurelie.delcont@anses.fr (A.D.C.); cristina.gastaldi@anses.fr (C.G.); 2 Inter Laboratory Comparison Consortium, European Union Reference Laboratory for Bee Health, 06902 Sophia Antipolis, France; eurl.bee@anses.fr

**Keywords:** diagnosis, inter-laboratory comparison, morphology, real-time PCR, honey bee, *Aethina tumida*, Small Hive Beetle

## Abstract

**Simple Summary:**

*Aethina tumida*, also called the Small Hive Beetle, is an insect that multiplies primarily in honeybee hives, causing honey losses and weakening colonies. It is native to sub-Saharan Africa and was introduced into different countries and continents over the last 20 years, posing a threat to beekeeping internationally. In case of introduction into a new area, officially approved laboratories (certified by government services) carry out analyses to confirm the outbreak. The reliability of the results is essential in the implementation of management measures. Therefore, a study was organised at the European level to compare the results between official laboratories for two types of methods, used routinely for the identification of *A. tumida*: morphological examination (form and structure) and DNA testing (genetics). The 22 participants analysed in a blinded way a panel of 12 samples (positive and negative samples). The results were very satisfactory, with the exception of one participant who encountered several anomalies for negative samples and especially for DNA tests, probably related to his inexperience with the method. This study proved the ability of laboratories and analytical methods to identify *A. tumida*, which is a key element in monitoring and managing this risk.

**Abstract:**

The Small Hive Beetle (*Aethina tumida* Murray, 1867) is an invasive scavenger of honeybees. Originally endemic in sub-Saharan Africa, it is regulated internationally in order to preserve the areas still free from this species. To ensure the reliability of official diagnoses in case of introduction, an inter-laboratory comparison was organised on the identification of *A. tumida* by morphology and real-time PCR. Twenty-two National Reference Laboratories in Europe participated in the study and analysed 12 samples with adult coleopterans and insect larvae. The performance of the laboratories was evaluated in terms of sensitivity and specificity. Sensitivity was satisfactory for all the participants and both types of methods, thus fully meeting the diagnostic challenge of confirming all truly positive cases as positive. Two participants encountered specificity problems. For one, the anomaly was minor whereas, for the other, the issues concerned a larger number of results, especially real-time PCR, which probably were related to inexperience with this technique. The comparison demonstrated the reliability of official diagnosis, including the entire analytical process of *A. tumida* identification: from the first step of the analysis to the expression of opinions. The performed diagnostic tools, in parallel with field surveillance, are essential to managing *A. tumida* introduction.

## 1. Introduction

The Small Hive Beetle (SHB), *Aethina tumida* Murray, 1867 (Coleoptera: Nitidulidae), is an invasive scavenger of honeybee colonies native to sub-Saharan Africa [1]. Part of its biological cycle takes place in *Apis mellifera* colonies and part in the soil. Adult beetles, attracted by the smell of the hive, enter it to reproduce. They lay masses of eggs in wood crevices or inside combs that hatch into larvae. The predatory larvae grow by feeding on bee brood, pollen and honey. Their faeces cause fermentation processes in the hive, making the honey unfit for human consumption. Once they have grown to a sufficient size, after a few weeks, the larvae leave the hive to begin their pupation in the soil. The development of larvae in the hive may cause significant damage for beekeeping, which can, in the most severe cases, result in loss of the entire bee colony and harvest losses [2]. Cases of infestation of bumblebee colonies (*Bombus spp.*) and of solitary bee nests were also reported, but data are still lacking on the impact of *A. tumida* on these species [3,4]. Outside host nests, adult SHBs can feed on alternative sources, such as fruits or foraging on flowers [4,5].

The SHB disperses naturally by flight over unknown distances (possibly more than 10 km) [6]. However, over the last twenty years, it was introduced to different continents outside its natural range [6,7]. Migratory beekeeping, globalisation and international trade play a major role in its spread over long distances [8]. Trade-in wax could facilitate SHB invasion [9]. Therefore, in order to protect *A. tumida*-free areas, infestation by the SHB is regulated at the international level and it is listed as a notifiable disease to the World Organisation for Animal Health (OIE) [10].

In the European Union (EU), the first case of introduction was reported in Portugal in 2004, following the import of a bee queen from Texas, United States [11]. Early detection and effective control measures enabled eradication [12]. Ten years later, in September 2014, the presence of SHB was confirmed in the region of Calabria, in southern Italy [13,14]. Control measures were set up immediately, but visits to the apiaries in the same areas demonstrated that *A. tumida* had spread in this region [15], where it can now be considered as established. So far, containment measures have prevented the spread of SHB to the rest of the EU, which remains free of this parasite. In order to limit the spread of SHB and further introductions into the EU, infestation by *A. tumida* is subject to compulsory surveillance and notification according to the “Animal Health Law”. Moreover, regulations apply to intra-EU trade and to imports from non-EU countries [16,17] to prevent SHB introduction.

Detection of *A. tumida* in apiaries relies mainly on inspection of honeybee colonies and installation of traps [18]. These two approaches enable the visual detection of adult coleopterans and larvae. Molecular analysis of hive debris is also an alternative way to detect the presence of SHB DNA [19], although the method needs to be validated in field conditions to better evaluate its sensitivity [3]. When suspicious specimens are detected, a differential diagnosis should be established with other beetles and insects living in the hive environment. Particularly, a distinction should be made with other beetles belonging to the Nitidulidae family, such as *Cychramus luteus* Fabricius, 1787 [20] and *Carpophilus lugubris* Murray, 1864 [21], which do not have a detrimental effect on bees but seek refuge in the hive and feed on pollen or debris. The larva of *A. tumida* can also be mistaken for larvae of the lesser wax moth, *Achroia grisella* Fabricius, 1794 (Lepidoptera: Pyralidae), as well as for the honeycomb moth, *Galleria mellonella* Linnaeus, 1758 (Lepidoptera: Pyralidae). These Lepidoptera are frequently found in colonies and on beekeeping equipment. They can cause damage to the combs of weak colonies and on frames that are not properly stored [22].

In case of suspicion, laboratory diagnosis is crucial to reliably and rapidly identify *A. tumida* and confirm the outbreak. This diagnosis is supported by the quality of the methods and by the competence of the laboratories officially approved to carry out analyses (i.e., certified by government services). Two types of methods are routinely used and recommended in the Manual of Diagnostic Tests and Vaccines for Terrestrial Animals of the OIE for the identification of *A. tumida* [7]: (i) morphological examination, which provides a result in a short amount of time through a low-cost technique and which is therefore particularly indicated for first-line diagnosis, and (ii) PCR, which is more sensitive and specific, and which is generally used as a second step.

In response to diagnostic issues and to ensure the quality of the analytical results obtained within the EU, the European Union Reference Laboratory (EURL) for Bee Health, located in the laboratory of the French Agency for Food, Environmental and Occupational Health and Safety (ANSES) in Sophia-Antipolis (France), organised an inter-laboratory comparison using morphology and molecular identification of *A. tumida* in October 2020. This was the first trial organised using these methods. All the EU National Reference Laboratories (NRLs) were invited to participate. Non-EU countries that were interested in the comparison were also included. The evaluation was supported by a blind analysis of a panel of 12 insect samples. The objective was to evaluate the sensitivity, specificity and accuracy of the results obtained by the laboratories with the analytical methods they routinely use for an official diagnosis.

## 2. Materials and Methods

### 2.1. Participating Laboratories

Twenty-two NRLs for Bee Health took part in the inter-laboratory comparison, 21 from EU member states and one from another European country (Table A4). Sixteen participants used morphological and PCR identification methods, whereas six used morphological identification only, depending on their diagnostic possibilities. Importantly, the latter have not yet implemented real-time PCR for the identification of *A. tumida*, and refer to another official laboratory, competent for this method, if confirmation is required.

### 2.2. Reference Methods for Panel Sample Characterisation

Two reference methods were used by the EURL to characterise and to check the homogeneity and the stability of the samples for comparison: (i) the morphological method (EURL procedure, also published in the OIE Manual), and (ii) the PCR method (EURL procedure, also published in the OIE Manual) [7,23,24]. The EURL is accredited by the French Accreditation Committee (COFRAC) for these two methods, in compliance with the international standard NF EN ISO/IEC 17025 on “General requirements for the competence of testing and calibration laboratories” [25]. Moreover, sequencing of the COI gene was also performed to determine or confirm the species of the panel specimens.

The morphological method consists of the visual examination of individuals (adults and/or larvae) with recording of specific morphological characteristics and, if necessary, comparison of the sample to be identified with a reference sample or detailed photographs. The identification relies on the assessment of eight criteria for the adult and three criteria for the larva (Table 1). A stereomicroscope (and/or magnifier) (minimum 40 × magnification) is used during the analysis. For the adult, if all the criteria are present, the final result of the examination is “positive”. For the larva, as the number of criteria is limited, the presence of all the criteria should be considered a “suspicion” and must always be confirmed by PCR. If at least one criterion is absent, the result of the analysis is “negative”. Finally, in some cases (e.g., damaged specimen) the result can be inconclusive because it is not possible to assess the presence of certain morphological identification criteria. In this situation, PCR identification must systematically be performed.

Real-time PCR was used to confirm specimen identification, using species-specific primers designed by Ward et al. [19] for the COI region of *A. tumida*. Briefly, specimens (adult or larva) are ground manually in an appropriate volume of phosphate buffer (1 mL for one adult specimen, 200 µL for one larva) after a rinse with the same buffer (as specimens are preserved in 70% ethanol). Eighty µL of the crushed suspension is used to extract DNA using a QIAamp DNA Mini kit (Qiagen, Hilden, Germany). The extracted DNA is eluted in 200 µL of elution buffer, according to the manufacturer’s recommendations and stored at -20 °C ± 5 °C until further analysis. The realtime PCR is performed in a 25 µL reaction system containing 2x SsoAdvanced Universal Probes Supermix (Bio-Rad, Marnes-La-Coquette, France) 12.5 µL, forward (5′-TCTAAATACTACTTTCTTCGACCCATCR-3′) and reverse (5′-TCCTGGTAGAATTAAAATATAAACTTCTGG-3′) primers (100 µM) 0.4 µL, TaqMan^®^ probe (5′-ATCCAATCCTATACCAACACTTATTTTGATTCTTCGGAC-3′) (50 µM) 0.05 µL, TaqMan^®^ Exogenous Internal Positive Control Mixture (Applied Biosystems, Waltham, MA, USA) 2.5 µL, 50× Internal Positive Control (IPC) DNA (Applied Biosystems, Waltham, MA, USA) 0.05 µL, nuclease-free H_2_O 4.1 µL and 5 µL of DNA template. The PCR runs consist of an initial step of 3 min at 95 °C, followed by 40 successive cycles of 10 s at 95 °C and 30 s at 60 °C. The reaction is run on a CFX96 RealTime PCR system (Bio-Rad, Marnes-La-Coquette, France). DNA detection is expressed in Ct values. Positive and negative controls are used in each DNA extraction and PCR session and in every run, the non-template controls and the positive controls should have the expected results. The success of the amplification and the absence of inhibition is verified by the result of an exogenous IPC. The specific limit of positivity was characterised during the validation process. Thus, it was defined that a result is positive when the Ct value (cycle threshold) is under 35.

Sequencing of the mitochondrial cytochrome oxidase subunit I (COI) gene was performed on specimens to determine or confirm the species. A fragment of 710 bp was amplified with universal primers LCO1499 (5′-GGTCAACAAATCATAAAGATATTGG-3′)/HCO2198 (5′-TAAACTTCAGGGTGACCAAAAAATCA-3′) [26]. The amplification products were sequenced by the Sanger method, using the two primers previously mentioned. From the consensus sequences, a query of the NCBI databases using a Basic Local Alignment Search Tool (blast.ncbi.nlm.nih.gov) was performed.

### 2.3. Selection of Inter-Comparison Samples

The panel included two types of samples: (i) positive samples, i.e., adult and larval specimens of *A. tumida* Murray species (Coleoptera, Nitidulidae family), and (ii) negative samples, i.e., adult and larva specimens of insect species other than *A. tumida.*

The adult *A. tumida* specimens (A-POS) were obtained experimentally in the confined laboratory of Fera Science, Ltd. in 2019 (York, UK), whereas the *A. tumida* larvae (L-POS) were collected in Maryland (Beltsville, MD, USA) in 2013.

The negative species were selected based on different criteria of interest:

- The fact that the coleopteran species belong to the Nitidulidae family (i.e., same family as *A. tumida*), and therefore have similar morphological and molecular characteristics, and/or,
- The fact that the species were likely to be found in the honeybee hive environment, and/or,
- The fact that the species presented morphological features close to *A. tumida*, and,
- The availability of specimens in sufficient numbers to constitute the panels and to carry out homogeneity and stability tests.

Thus, four different adult species were selected (Table 2): *Tenebrio molitor* (A-NEG1), *Alphitobius diaperinus* (A-NEG2), *Epuraea luteola* (A-NEG3), and *Cryptolaemus montrouzieri* (A-NEG4). The negative larvae belonged to three different species: *Tenebrio molitor* (L-NEG1), *Galleria mellonella* (L-NEG2), and *Carpophilus dimidiatus* (L-NEG3).

Each batch of samples was characterised at the EURL by the two independent accredited methods described in Section 2.2 (morphology and PCR), and sequencing of the COI gene was performed for the negative specimens to determine the species.

In total, the panel distributed to the participants contained 12 samples for comparison: two positive and four negative adult coleopterans, two positive and three negative insect larvae, and a “lure”, which was either positive or negative. The “lure” sample was not evaluated and was included in the panel to limit the risk of collusion.

Each sample in the panel consisted of one specimen (one adult or one larva) which was packaged in a 2 mL micro-tube, filled with non-denatured ethanol 70%. During the packaging process, the integrity of each specimen was visually controlled. When detected, damaged specimens (e.g., specimens with broken legs, incomplete abdomen segments or broken antennae) were excluded from the assay. The samples were stored at room temperature until shipment.

### 2.4. Homogeneity and Stability Tests

The homogeneity study was performed for all the sample batches packed in micro-tubes from December 2019 to January 2020. Considering the characteristics of the batches (origin of the specimens particularly) and the number of specimens prepared, the number of samples to be taken was defined specifically for each batch.

For the A-NEG2 and the L-POS batches, which were collected in the field and with a risk of non-homogeneity, the protocol strictly followed the recommendations of the international standard NF ISO 13528 on “Statistical methods for use in proficiency testing by interlaboratory comparisons” [27], which specifies that 10 samples are to be randomly taken per batch and analysed in duplicate. For the other batches, the risk of non-homogeneity was considered low, taking into consideration the following aspects:

- The specimens from batches A-POS, A-NEG1, L-NEG1, A-NEG4 were produced artificially in a controlled environment;
- The specimens from batches L-NEG3 and A-NEG3 were provided and previously analysed by specialised entomologists;
- The EURL produced the *G. mellonella* larvae (L-NEG2) artificially from combs coming from the ANSES apiary in Sophia-Antipolis (territory officially free from *A. tumida* at sampling date in 2019);
- The results of the homogeneity tests of a preliminary study carried out in 2018 were satisfactory for *A. tumida* specimens (A-POS) and *T. molitor* specimens (A-NEG1 and L-NEG1), of the same origin;
- The specimens were visually controlled when the tubes were prepared.

For these samples, the sampling strategy was designed according to the International Laboratory Accreditation Cooperation (ILAC) recommendation [28] and based on the number of samples prepared (Table A1). This protocol made it possible to reduce the sample size and to address the lack of availability of some specimens. The tubes were analysed, first by morphological examination, and then by molecular identification. The samples were analysed in duplicate by PCR (i.e., by analysing two collections per sample). All the results met the expected values of 100% positive results for positive samples, and 100% negative results for negative samples, which validated the homogeneity study.

Results from analyses carried out on samples coming from the EURL entomological collection and from preliminary data showed that the samples were stable in 70% ethanol at room temperature for several years. In order to confirm the stability of the samples during the assay, tests were also performed at the deadline for reporting the results. In compliance with standard NF ISO 13528, three samples from each batch were randomly taken and analysed by the two reference methods of the comparison (morphology and PCR, with a duplicate analysis for the latter). The results were consistent with the expected values, which confirmed the stability of the samples.

### 2.5. Process for the Interlaboratory Comparison

The trial was organised in compliance with the quality requirements described in international standard NF EN ISO/IEC 17043 on “General requirements for proficiency testing” [29]. The samples were packed and shipped between the EURL and NRLs in compliance with the International Air Transport Association (IATA) regulation for shipping and handling dangerous goods.

Each participating laboratory was anonymously coded with a 1- or 2-digit random number to ensure confidentiality of the results. Each sample included in the blind-test panel was coded with the attribution of a random number between 1 and 12. Participating laboratories received inter-comparison samples with a laboratory code on each tube. After receiving the package, the laboratories were required to store the samples at room temperature until analysis and to send back their results within 24 days after the shipment date of the panel samples.

They were asked to report the qualitative results corresponding to one or both of the methods used to analyse the samples. For morphology, three types of values could be rendered: “positive” (or “suspicion” for larvae), “negative” and “inconclusive” (i.e., impossibility to confirm the presence or absence of certain characteristics). For PCR, there were also three modalities: “positive”, “negative” and “inhibited” (i.e., PCR reaction inhibited).

In order to conclude on the results of different analyses performed (morphology and PCR), guidelines were given for expressing opinions. They were based on the decision rules described in Table 3. In particular, these opinions allow for consideration of cases where the results are “inconclusive” in morphology or/and “inhibited” in PCR. These opinions are important for health authorities who must then decide what control measures to implement based on the analytical results.

For each analytical method, three criteria of performance were assessed by adding the scores obtained for the different samples:

1. Sensitivity, i.e., the ability of the laboratory to give a positive result for a positive sample [30];
2. Specificity, i.e., the ability of the laboratory to give a negative result for a negative sample [30];
3. Accuracy, i.e., the closeness of agreement between the obtained results and the assigned values, definition adapted from international standard NF EN ISO 16140 [31].

The analytical conclusion was not evaluated but was analysed as complementary information.

### 2.6. Results Evaluation

First, the coherence of the inter-laboratory comparison was checked by analysing the raw results of all the participants. In a second step, the EURL assessed the individual performance of the laboratories and the overall performance of the NRL network.

The assigned values were designated beforehand in compliance with international standard NF ISO 13528. They were based on expert judgement and on the knowledge of the origin and the preparation of the specimens. Moreover, the status of the samples was controlled according to the two independent reference methods (morphology and PCR).

For each analytical method, the results of the participants were evaluated in compliance with international standard NF ISO 13528, by scoring each single result based on the assigned value Then, the three criteria for performance (sensitivity, specificity and accuracy) were assessed by summing, for each method and participant, the scores obtained for the different samples (Table A2). For each performance criterion, a total score of “0” was considered “satisfactory”, whereas a total score of “1” was considered a “warning signal” and a total score “≥2” was considered an “action signal”, requiring the implementation of corrective actions.

The overall performance of the network was assessed taking into account all the results of the laboratories. The rate of sensitivity was calculated, corresponding to the percentage of true positive results obtained by all participating laboratories out of the total number of positive samples distributed and evaluated (i.e., with a positive assigned value). The rate of specificity was also evaluated corresponding to the percentage of true negative results obtained by all participating laboratories out of the total number of negative samples distributed and evaluated (i.e., with a negative assigned value). Finally, the rate of accuracy corresponded to the percentage of accurate results (true positives and true negatives) obtained by all participating laboratories out of the total number of samples distributed and evaluated. We calculated these rates for the two different techniques and for the analytical conclusion, applying the formulas given by international standard NF EN ISO 22117 on “Microbiology of the food chain-Specific requirements and guidance for proficiency testing by interlaboratory comparison” [30].

### 2.7. Information on Analytical Methods Employed by the Participants

The participants were asked about the analytical methods they used for the trial. They had to specify the reference of their methods (i.e., the official method disseminated by the EURL that is also published in the OIE Manual or another one, which could for example be published in the literature). If they used the EURL reference method for morphology, detailed results of the examination of each criterion (Table 1) had to be rendered. For PCR, laboratories were requested to report technical information on their extraction method, amplification kit and thermocycler used. In addition, they were asked to provide the Ct value obtained for each sample.

## 3. Results

### 3.1. Individual Laboratory Performance for the Morphological Identification of A. tumida

**Figure A1:**
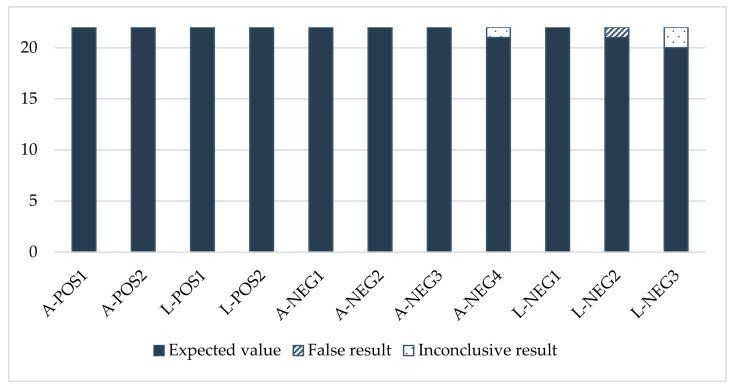
Consistency of the morphology results.

The results showed good consistency for all samples in the panel (Figure A1). They complied overall with the expected values. We observed a few disparities for three negative samples (A-NEG4, L-NEG2 and L-NEG3), but they concerned a limited number of laboratories.

Sensitivity was satisfactory for all the participants (score of 0) (Table A3). However, two laboratories encountered problems regarding specificity. Laboratory No. 11 gave a positive result for a negative larva sample (L-NEG2), leading to a score of 2, considered an “action signal”. Laboratory No. 6 gave an inconclusive result for the negative larva sample L-NEG3, leading to a score of 1, considering the fact that one morphological criterion was mis-assessed. This score was considered a “warning signal”.

Laboratories No. 9 and No. 16 also gave inconclusive results for samples A-NEG4 and L-NEG3, respectively as they were not able to assess the presence of a morphological criterion. However, the results were considered acceptable, given the explanation reported by the participants and the relevant results of the evaluation for the other criteria assessed. Thus, they were given a score of 0 for these samples.

### 3.2. Individual Laboratory Performance for PCR Identification of A. tumida

**Figure A2:**
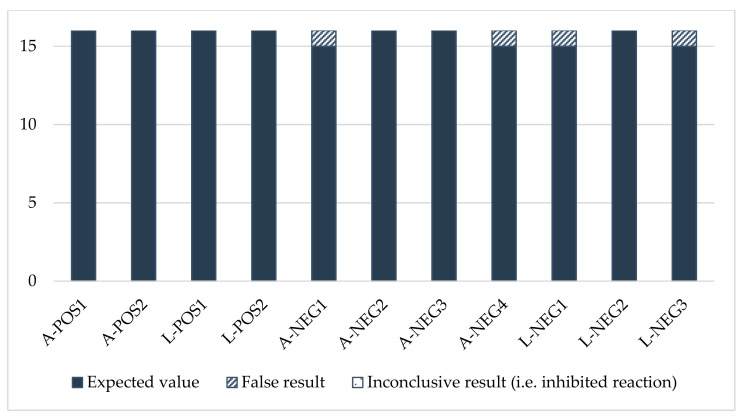
Consistency of the PCR results.

The PCR results also showed good consistency (Figure A2). With the exception of one laboratory, they complied with the expected values.

Sensitivity was satisfactory for all the participants (score of 0) (Table A3). One laboratory experienced problems regarding specificity. Laboratory No. 11 gave positive results for two negative adult samples (A-NEG1 and A-NEG4) and two negative larva samples (L-NEG1 and L-NEG3), leading to a total score of 8, considered an “action signal”.

### 3.3. Individual Laboratory Performance for Expressing Opinions

The instructions specified that a conclusion had to be given for each sample, based on the results of the analyses carried out (Table 2).

**Figure 1:**
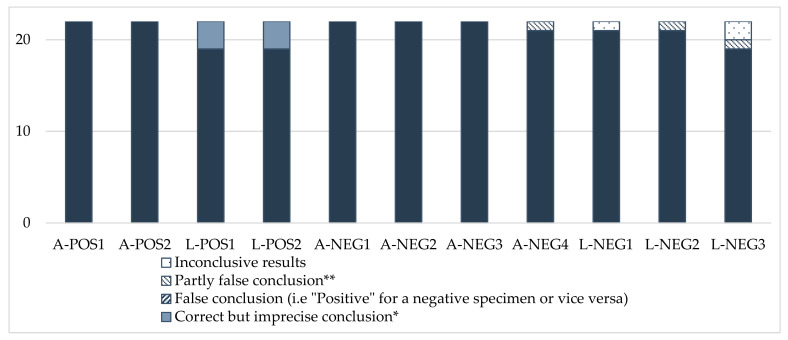
Analytical conclusions. * For larvae: “Positive identification of the Small Hive Beetle, *A. tumida*”, instead of “Suspected identification of the Small Hive Beetle, *A. tumida*. Further analysis is required to ascertain the identification”, as precisely expected in this case, where identification was based on morphology only. ** “Suspected, further analysis required in confirmation”, whereas the specimen was negative.

The analytical conclusions were overall satisfactory for the positive and negative samples (Figure 1). However, it should be noted that three participants (Nos. 7, 8 and 20) that only used morphological identification concluded, for the positive larvae, “positive identification of SHB, *Aethina tumida*”, whereas the expected answer was more precisely: “suspected identification of SHB, *Aethina tumida*. Further analysis is required to ascertain the identification”. In this case, molecular identification is necessary to confirm the morphology result. For negative specimens, three laboratories (Nos. 6, 11 and 16) could not conclude with certainty on some samples because of the anomalies observed with their analytical results.

### 3.4. Overall Performance of the Participants

The overall performance of the participants for morphological analysis reached a rate of 100% for sensitivity, 98.7% for specificity, and an overall accuracy rate of 99.2% (Table 4). The PCR results were also very satisfactory, with 100% sensitivity, 97.4% specificity and 98.3% accuracy. For the analytical conclusion, the rates ranged from 93.2% to 96.1%, due to the minor anomalies observed for the positive larvae, and to the problems regarding specificity identified for some negative specimens.

### 3.5. Performance of the Methods Used for the Official Diagnosis of A. tumida

The 22 participants used the EURL protocol for morphological identification, which is also published in the OIE Manual [7,23].

Out of 16 participants who performed the PCR method to confirm the morphology results, 14 participants used the EURL protocol based on the publication by Ward, et al. [19], which is also referenced in the OIE Manual [7]. However, none of them used the same experimental conditions as those of the EURL in terms of the combination of extraction kit, amplification kit and thermocycler used. The Ct values obtained for the adult SHB specimens ranged from 21.3 to 30.8, and for the larva SHB specimens from 16.4 to 30.5. As mentioned above, one participant (No. 11) reported four positive results on negative specimens (Ct values ranging from 30.9 to 34.9). The other two participants (No. 10 and No. 18) used the PCR method described by Silacci, et al. [32]. The Ct values obtained for the adult SHB specimens ranged from 22 to 39.8, and for the larva SHB specimens from 16.4 to 30.5. The two participants who used this method had different results on the negative specimens. For one of them, no amplification was observed, while for the other Ct values ranging from 34.8 to 40.2 were reported. However, the analytical conclusion of this participant was in accordance with the expected identification. The Ct differences observed in the participants may be due to more or less efficient rinsing of the specimens and to the DNA extraction method. The evaluation of the participants was based on the complete process.

## 4. Discussion

In general, the surveillance of exotic diseases, also called epidemio-vigilance, aims to detect diseases as early as possible [33,34]. Thus, in a country (or a zone) free from *A. tumida*, the objective is usually to detect any outbreak at an early stage in order to increase the chances of eradication [18]. In this context, it is essential that the performance of official laboratories and tests are appropriate for this intended surveillance and/or control objectives [35]. Sensitivity and specificity are the two main criteria for the evaluation of this performance [33]. For an exotic pathogen and a high-risk disease, the need to have a high level of sensitivity is even more essential because the challenge is to confirm all truly positive cases as positive, and thus avoid missing the detection of a possible introduction [34]. The results of the inter-laboratory comparison showed that sensitivity was satisfactory (overall performance of 100%) for all 22 participants and for both types of methods, morphology and PCR), therefore fully meeting this diagnostic challenge.

Specificity is also an important criterion to eliminate the possibility of false-positive results and thus, to avoid false health alerts and unnecessary destruction of honeybee colonies [34]. The overall performance of the network was 98.9% for morphology and 97.4% for PCR. Two laboratories encountered problems with specificity. Laboratory No. 6 obtained a “warning signal” for their morphological identification (specificity score of 1) due to a misjudgement of criterion 3 on sample L-NEG3, corresponding to a larva of *C. dimidiatus.* The “spines” are indeed absent in this species. Following the inter-laboratory comparison, the laboratory explained that they damaged the specimen when they took it out of the tube. The specimen was in fact stuck to the wall of the tube, and during removal, it was crushed and cut into two pieces. Therefore, the participant could not properly evaluate the morphological criteria. Analysing the cause showed that this anomaly has a very limited impact and can be evaluated as minor. However, this highlights that specimen handling is a critical point in the analysis. To detach the specimens from the tube, ethanol (70%) can be poured into the tube to loosen the specimen from the wall, possibly by closing the tube and shaking it gently. In addition, in case of mishandling, PCR remains the tool of choice to confirm the result of the morphological analysis. Laboratory No. 11 experienced specificity issues resulting in an “action signal” in both microscopy and PCR (specificity scores of 2 and 8, respectively). The larva sample L-NEG 2 was found to be positive after morphological identification, whereas the expected value was negative (and the PCR result was also negative). The specimen was a wax moth larva with “false legs” on its posterior segments (criterion 2 absent) and had no dorsal “spines” (criterion 3 absent). Moreover, the PCR results were positive for the four negative samples A-NEG1, A-NEG4, L-NEG1 and L-NEG3, whereas their morphological results were negative. The participant indicated that they were inexperienced and that they performed the PCR method for the first time. Furthermore, the participant mentioned that the method used did follow the guidelines described by the EURL; however, the combination of extraction method and reagents used for PCR had not yet been tested against the reference materials. The evaluation of sensitivity had therefore not been defined under the conditions described by this participant. These results demonstrate that the validation and adoption stages of the methods are crucial to ensure the reliability of the analytical results. These steps include participation in training and the use of reference materials [36]. In the framework of the EU network of NRLs, the EURL for Bee Health has organised two training courses to support laboratories in the adoption of the reference methods for *A. tumida* identification: one in 2014 on the morphological identification of *A. tumida*, and a more general one in 2016 on PCR diagnosis of honeybee diseases. Following this training, reference specimens and PCR-positive controls were distributed to laboratories. The inter-laboratory comparison organised in 2020 proved that the adoption process was an overall success for the participants, with the exception of one laboratory for which support (e.g., additional training) will be given.

The results of the comparison confirmed the reproducibility of the official methods for the morphological and molecular identification of *A. tumida*. All the participants strictly used the EURL reference method (OIE method) for morphological identification [7,23]. This method is suitable and reliable for routine official diagnosis in reference laboratories for bee diseases, but not necessarily for specialists when it comes to morphological identification of Coleoptera, which is a field that in itself requires specific skills and equipment. The precise morphological identification of *Aethina* species relies on the use of identification keys and on the expertise of entomologists specialising in Coleoptera, and in the family Nitidulidae itself [37]. Thus, the method may have limited performance for accurate identification of *Aethina* spp. in a research setting, but is highly suitable for diagnostic purposes and meets the operational requirements of international reference laboratories [35]. Diagnosis through PCR technology can be used to confirm the precise identification of the species in case of doubt. However, when a first case is detected in a territory, it would be advisable to implement PCR systematically to avoid any uncertainty or doubt.

The 87% of the laboratories that applied the PCR method corresponding to the EURL reference method (i.e., based on Ward, et al. [19] and also referenced by the OIE) used different types of experimental conditions. Eight different DNA extraction and eleven different PCR mix methods were used by the participating laboratories. This led to the use of 22 different protocols. The diversity of the experimental conditions used in this trial did not, however, affect the PCR result (93% satisfactory results), thus demonstrating the robustness of this method.

The comparison also included a part concerning the expression of opinions. The outcomes were satisfactory and correlated well with the analytical results. This aspect is crucial because it is on this basis that health authorities make decisions on the surveillance or control measures to be applied. However, it should be mentioned that while laboratory diagnostic performance is an important component of *A. tumida* surveillance, it is also essential to combine it with good sampling and thus with high sensitivity of field detection methods [38]. The OIE Manual describes several approaches based on the visual inspection of colonies and the use of traps [7], but they still need to be standardised. In addition, there is a lack of knowledge on their performance level in low-infested colonies. The sensitivity of these field methods may also vary depending on seasons, climate, environment conditions and colony strength [6,39]. Concerning the molecular approach, a method for the detection and the identification of *A. tumida* DNA in hive debris is described [19]. However, again, validation data are lacking to characterise its performance in the field in the context of low infestation rates [3].

Lastly, the assay demonstrated the feasibility of an inter-laboratory comparison organised at the international level on the official diagnosis of *A. tumida*, which is to our knowledge the first in this field. The comparison aimed to include the whole analytical process: the first step of morphological examination of the specimen, the PCR step in confirmation, and finally the expression of opinions. The samples were prepared and stored in ethanol (70%) at room temperature, as expected in routine analysis. The objective was to match the reality of analyses as closely as possible. Thus, the comparison allowed for inconclusive results and therefore a specific scoring system was established to evaluate participant performance, taking into account that specimens could be damaged or that the PCR could be inhibited. In addition, this original scoring system, based on the recommendations of international standard NF ISO 13528 [27], made it possible to prioritise the performance results and translate them into action or warning signals, similar to the calculation of the z-score, which is applied for quantitative trials.

The availability of relevant specimens is a difficulty in organising this type of trial. Specimens of *A. tumida* can quite easily be collected in infested areas or obtained experimentally. The cost to recover these specimens remains significant, however. The most difficult part is to get an appropriate amount and variety of negative specimens to include in the test. It would have been interesting to include more specimens of Nitidulidae, and particularly, some more morphologically close to *A. tumida*, or even other species of beetles frequently found in hives. The challenge is to have a sufficient number of them to constitute all the panels and to perform the homogeneity and stability tests. This practical constraint makes it necessary to adopt the recommendations of NF ISO 13528 for testing the homogeneity of the samples for comparison. Like in the field of plant health comparisons, a relevant risk analysis has to be performed to reduce the number of samples to be analysed according to this standard [40]. This was carried out specifically for each batch of samples, taking into consideration appropriate data available. Finally, it should be mentioned that one of the critical points for the organisation of this type of comparison, including morphological analysis, is the preparation of the samples. Careful examination of the specimens is clearly necessary to eliminate, as far as possible, those that are damaged and for which evaluation of all the criteria may not be possible.

## 5. Conclusions

In this study, we compared the performances of laboratories in identification methods of *Aethina tumida* across 22 European countries. This inter-laboratory comparison is, to our knowledge, the first organised at the international level on the official diagnosis of *A. tumida*.

It demonstrated the reliability of the reference methods, including the whole analytical process (morphology, PCR confirmation and opinions). These diagnostic tools are essential in the surveillance and management of *A. tumida* introductions in countries where its presence has not yet been shown and where early detection is crucial.

Avenues for improvement were identified, especially the inclusion of Coleoptera species more similar to *A. tumida* among the samples to be blind tested. However, in view of the difficulties encountered, in particular in constituting the sample panel, the study proved the feasibility of organising an international inter-laboratory comparison in this field.

## Figures and Tables

**Table 1:** Morphological identification of *Aethina tumida* Murray–Criteria for adult and larva diagnosis [7,23].

Criteria	Adult Form
1	Body divided into three parts: head, thorax and abdomen.
2	Three pairs of legs.
3	Presence of elytra ^1^.
4	Elytra not covering the entire abdomen: some abdominal segments are apparent in dorsal view.
5	Overall uniform body colour (no spots), light brown to black ^2^.
6	Antenna tips with compact, almost rounded club ends. The three terminal articles of the antennae, corresponding to the “clubs” ^3^, are narrowed between them, and their length is almost equal to their width.
7	Sharp latero-posterior tips of the pronotum ^4^.
8	Dimensions. Length: 4 to 7 mm (±1 mm). Width: 3 mm (±1 mm).
	**Larval form**
1	Three pairs of legs, one on each of the anterior segments, corresponding to the larva thorax.
2	All of the abdominal segments are bare and have no false legs (also called pseudopods) on their ventral part.
3	From the mesothorax ^5^, presence on each segment, of two dorsal tubercles on either side of the midline. These tubercles are finished with a short fine seta. They look like “spines”.

^1^ Elytra are sclerotised (meaning thickened) forewings covering the hind wings at rest in beetles and some other insects. ^2.^ Depending on the maturity of the specimen, the colour of adult *A. tumida* varies from light brown/red brown after emergence to dark brown to black when fully mature. ^3^ In some beetle families, such as the Nitidulidae, the terminal articles of the antennae are larger and club-shaped. ^4^ The pronotum is the dorsal part of the first segment of the thorax. The first segment of the thorax, called the «prothorax», carries the first pair of legs on the ventral side. ^5^ The mesothorax corresponds to the second thoracic segment of the larva. It carries the second pair of legs. The prothorax corresponds to the first thoracic segment; it does not have a tubercle, its dorsal part (tergum) is sclerotised.

**Table 2:** Nature and origin of the different specimens included in the sample panel for comparison. (Se: Sensitivity; Sp: Specificity; Ac: Accuracy).

Sample Code	Species			Origin	Criteria Evaluated
**ADULTS**					
A-POS	*Aethina tumida* (Murray, 1867)	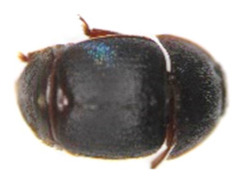	Coleoptera Nitidulidae Nitidulinae	Specimens obtained experimentally in the confined laboratory of Fera Science, Ltd. in 2019	Se, Ac
A-NEG1	*Tenebrio molitor* (Linnaeus, 1758) “Mealworm Beetle”	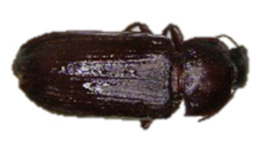	Coleoptera Tenebrionidae Tenebrioninae	Specimens obtained in 2018 by the company MICRONUTRIS providing insects for human consumption	Sp, Ac
A-NEG2	*Alphitobius diaperinus* (Panzer, 1797)	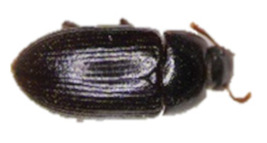	Coleoptera Tenebrionidae Tenebrioninae	Specimens collected in 2018 on the frame lid of honeybee hives located near a rabbit farm in Vendée (France)	Sp, Ac
A-NEG3	*Epuraea luteola* (Erichson, 1843)	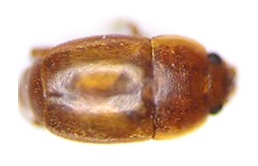	Coleoptera Nitidulidae Epuraeinae	Specimens collected in 2018 on *Citrus sinensis* in Corsica (France) and provided by the Plant Health Laboratory (ANSES, Montpellier, France)	Sp, Ac
A-NEG4	*Cryptolaemus montrouzieri* (Mulsant, 1853) “Ladybug”	* 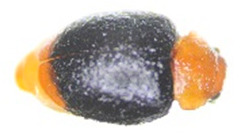 *	Coleoptera Coccinellidae Coccinellidae	Specimens obtained in 2019 by the company BIOLINE AGROSCIENCES providing insects for biological control in the plant field	Sp, Ac
**LARVAE**					
L-POS	*Aethina tumida* (Murray, 1867)	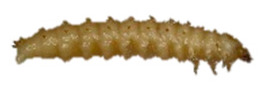	Coleoptera Nitidulidae Nitidulinae	Specimens collected in 2013 in Maryland (United States) and provided by J.S. Pettis	Se, Ac
L-NEG1	*Tenebrio molitor *(Linnaeus, 1758) “Mealworm beetle”	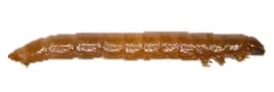	Coleoptera Tenebrionidae Tenebrioninae	Specimens obtained in 2018 by the company MICRONUTRIS providing insects for human consumption	Sp, Ac
L-NEG2	*Galleria mellonella*(Linnaeus, 1758) “Wax moth”	* 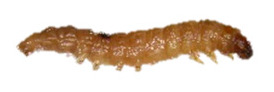 *	Lepidoptera Pyralidae Galleriinae	Specimens obtained experimentally in 2019 by the EURL, from hive frames coming from the EURL apiary and naturally infested by wax moth	Sp, Ac
L-NEG3	*Carpophilus dimidiatus* (Fabricius, 1792) “Sap Beetle”	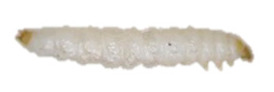	Coleoptera Nitidulidae Carpophilinae	Specimens collected in 2018 on crops (*Prunus dulcis*) in Côte-d’Or (France), provided by the Plant Health Laboratory (ANSES, Montpellier, France)	Sp, Ac

**Table 3:** Decision rules for expressing opinions, taking into consideration morphology and PCR results.

Morphology Result	PCR Result			
	*Analysis Not Performed*	*Positive*	*Negative*	*Inhibited*
**ADULT**				
*Positive*	(1)	(1)	(3)	(1)
*Negative*	(2)	(3)	(2)	(2)
*Inconclusive*	(4)	(1)	(2)	(4)
**LARVA**				
*Suspicion*	(3)	(1)	(2)	(4)
*Negative*	(2)	(3)	(2)	(2)
*Inconclusive*	(4)	(1)	(2)	(4)
**Opinion**				
(1) Positive identification of the Small Hive Beetle, *A. tumida*. (2) Negative identification of the Small Hive Beetle, *A. tumida*. (3) Suspected identification of the Small Hive Beetle, *A. tumida*. Further analysis is required to ascertain the identification. (4) Inconclusive result of *A. tumida* identification.				

**Table 4:** Overall performance of the official laboratory network.

Predicted Result	Morphology		PCR		Analytical Conclusion	
	Compliant Results	Non-Compliant Results	Compliant Results	Non-Compliant Results	Compliant Results	Non-Compliant Results
Positive	88	0	88	0	88	82
Negative	152	2	150	4	148	6
Sensitivity	100% (88/88)		100% (88/88)		93.2% (82/88)	
Specificity	98.7% (152/154)		97.4% (150/154)		96.1% (148/154)	
Accuracy	99.2% ((88 + 152)/(88 + 154))		98.3% (88 + 150)/(88 + 154)		95.0% (88 + 148)/(88 + 154)	

## Appendix A. Information on Homogeneity Tests

**Table A1:** Sampling strategy for homogeneity tests and results.

Code	Species	Number of Samples Prepared	Number of Samples Tested	Results
A-POS	*Aethina tumida*	115	10	100% Pos.
A-NEG1	*Tenebrio molitor*	60	7	100% Neg.
A-NEG2	*Alphitobius diaperinus*	60	10	100% Neg.
A-NEG3	*Epuraea luteola*	50	5	100% Neg.
A-NEG4	*Cryptolaemus montrouzieri*	60	7	100% Neg.
L-POS	*Aethina tumida*	115	10	100% Pos.
L-NEG1	*Tenebrio molitor*	75	7	100% Neg.
L-NEG2	*Galleria mellonella*	60	7	100% Neg.
L-NEG3	*Carpophilus dimidiatus*	60	7	100% Neg.

## Appendix B. Scoring Individual Performance

**Table A2:** Methodology of the score evaluation.

**Step 1–Scoring of Each Single Results, According to the Following Process**	
***Score of “0”***	Results that matched the assigned values exactly: - “Positive” result of the participant for a positive sample, - “Negative” result for a negative sample, - Or “inconclusive” result instead of positive or negative as assigned values, AND with justification (i.e., explanations and if possible, photos) regarding the state of the damaged sample.
***Score of “1”***	Results that did not match the assigned values exactly: - For morphological analysis, “inconclusive” result instead of positive or negative (as assigned values) AND without any justification regarding the state of the sample, - For PCR: “Inhibited” result instead of positive or negative (as assigned values).
***Score of “2”***	Results that did not match the assigned values: - “Positive” result of the participant instead of negative, - Or “Negative” result instead of positive.
**Step 2–Assessment of the three criteria for individual performance**	
***Sensitivity***	Sum of the scores obtained for all the positive samples of the panel.
***Specificity***	Sum of the scores obtained for all the negative samples of the panel.
***Accuracy***	Sum of the scores obtained for all the samples of the panel (positive and negative).
**Step 3–Final performance evaluation**	
For each performance criterion: - A total score of “0” is “Satisfactory”, - A total score of “1” is a “warning signal”, - A total score “≥ 2” is an “action signal”, requiring the implementation of corrective actions.	

## Appendix C. Detailed Results of the Participants

**Table A3:** Individual performance scores for morphology and PCR.

	Morphology			PCR		
Lab Code	Acc ^1^	Se ^2^	Sp ^3^	Acc ^1^	Se ^2^	Sp ^3^
**1**	0	0	0	0	0	0
**2**	0	0	0	0	0	0
**3**	0	0	0	0	0	0
**4**	0	0	0	0	0	0
**5**	0	0	0	0	0	0
**6**	**1**	0	**1**	0	0	0
**7**	0	0	0	NP	NP	NP
**8**	0	0	0	NP	NP	NP
**9**	0	0	0	0	0	0
**10**	0	0	0	0	0	0
**11**	**2**	0	**2**	**8**	0	**8**
**12**	0	0	0	NP	NP	NP
**13**	0	0	0	0	0	0
**14**	0	0	0	0	0	0
**15**	0	0	0	0	0	0
**16**	0	0	0	0	0	0
**17**	0	0	0	NP	NP	NP
**18**	0	0	0	0	0	0
**19**	0	0	0	0	0	0
**20**	0	0	0	NP	NP	NP
**21**	0	0	0	0	0	0
**22**	0	0	0	NP	NP	NP

^1^ Accuracy; ^2^ Sensitivity; ^3^ Specificity. NP = Not performed. A total score of **0** correspond to satisfactory/acceptable results, a score of **1** to a warning signal, and a score ≥ **2** to an action signal. Unsatisfactory or partially satisfactory scores (i.e., “warning signal” or “action signal”) are highlighted in bold.

## Appendix D. Members of the ILC Consortium

**Table A4:** Complete membership of the ILC Consortium (names listed in alphabetical order).

Name	Institute	Email
Rosen Aleksandrov	National Diagnostic and Research Veterinary Medical Institute, Aquaculture, Fish and Bee Diseases, NRL for Bee Diseases, 1040 Sofia, Bulgaria	rosen_angelov1989@abv.bg
Gabriela Chioveanu	Institute for Diagnosis and Animal Health, NRL for Animal Health, 50557 Bucharest, Romania	gabriela.chioveanu@idah.ro
Mary Coffey	Department of Agriculture, Food and the Marine, Plant Health Laboratories, W23 X3PH Celbridge, Ireland	MaryF.Coffey@agriculture.gov.ie
Benjamin Dainat	Agroscope Liebefeld, Swiss Bee Research Centre, 3003 Bern, Switzerland	benjamin.dainat@agroscope.admin.ch
María Pilar Fernández Somalo	Laboratorio Central de Veterinaria, Ministerio de Agricultura, Pesca y Alimentación (Gobierno de España), D.G. Sanidad de la Producción Primaria, S.D.G. Sanidad e Higiene Animal y Trazabilidad, 28110 Algete (Madrid), Spain	mfsomalo@mapa.es
Miriam Filipova	Department of Molecular Biology and Epizootiology, Veterinary and Food Institute in Dolny Kubin, State Veterinary and Food Institute, NRL for Honeybee Health, 026 01 Dolny Kubin, Slovakia	miriam.filipova@svpu.sk
Heather Graham	Wageningen Bioveterinary Research, Wageningen University and Research, NRL for Bee Diseases, 8221 RA Lelystad, The Netherlands	heather.graham@wur.nl
Anna Granato	Istituto Zooprofilattico Sperimentale delle Venezie, NRL for Honey Bee Health, 35020 Legnaro (PD), Italy	agranato@izsvenezie.it
Sirpa Heinikainen	Kuopio laboratory, Veterinary Bacteriology and Pathology Unit, Laboratory and Research Division, Finnish Food Authority, 70210 Kuopio, Finland	sirpa.heinikainen@ruokavirasto.fi
Hemma Köglberger	Department for Apiculture and Bee protection, Ages-Austrian Agency for Health and Food Safety, 1220 Vienna, Austria	hemma.koeglberger@ages.at
Nóra Krejczinger	National Food Chain Safety Office, Food Chain Safety Laboratory Directorate, NRL of Parasitology, Fish and Bee Diseases, 1095 Budapest, Hungary	krnori@gmail.com
Merle Kuus	Veterinary and Food Laboratory, 51006 Tartu, Estonia	merle.kuus@vetlab.ee
Severine Matthijs	Sciensano, Enzootic, Vector-borne and Bee Diseases, 1180 Brussels, Belgium	severine.matthijs@sciensano.be
Konstantinos Oureilidis	Veterinary Laboratory of Kavala, Directorate of Veterinary Center of Thessaloniki, Ministry of Rural Development and Food, 64012 Kavala, Greece	ktekav@yahoo.gr
Zanda Ozolina	Parasitology group, Laboratory of Microbiology and Pathology, Institute of Food Safety, Animal Health and Environment “BIOR”, LV-1076, Riga, Latvia	zanda.ozolina@bior.lv
Metka Pislak Ocepek	National Veterinary Institute, Veterinary Faculty, University of Ljubljana, Laboratory for Bee Health Care, Gerbičeva 60, 1000 Ljubljana, Slovenia	metka.pislakocepek@vf.uni-lj.si
Marc Oliver Schäfer	Friedrich Loeffler Institut-Germany, NRL for Bee Diseases, 17493 Greifswald-Insel Riems, Germany	marc.schaefer@fli.de
Emilia Semberg	Swedish University of Agricultural Sciences, Ecology, NRL for Honey Bee Health, 75007 Uppsala, Sweden	emilia.semberg@slu.se
Ivana Tlak Gajger	Laboratory for Honeybee Diseases APISlab, Department for Biology and Pathology of Fish and Bees, Faculty of Veterinary Medicine, University of Zagreb, 10 000 Zagreb, Croatia	ivana.tlak@vef.hr
Maria José Valério	Patologia Apícola, Patologia, Instituto Nacional de Investigacao Agraria e Veterinaria, I. P., 2780-157 Oeiras, Portugal	mjose.valerio@iniav.pt
Sarka Vyroubalova	Department of Pathological Morphology, State Veterinary Institute Olomouc, NRL for Honeybee Health, 77900 Olomouc, Czech Republic	svyroubalova@svuol.cz
